# Supplementary material for: Explainable, federated deep learning model predicts disease progression risk of cutaneous squamous cell carcinoma
Source: NPJ Precis Oncol. 2025 Jun 28;9:205. doi: 10.1038/s41698-025-00997-4 (PMC12206231; doi:10.1038/s41698-025-00997-4)
Supplement: Supplementary file 1 — Supplementary material [file 41698_2025_997_MOESM1_ESM.pdf]

Supplementary material

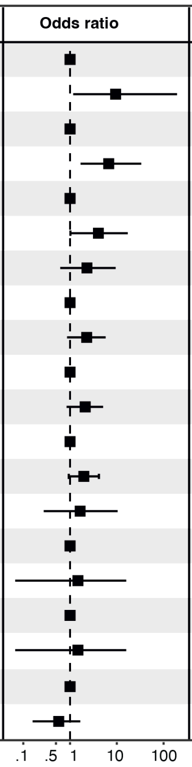

Variable		N	OR (95% CI, univ.)	p-value (univ.)
**Vascular invasion**	No	153		
	Yes	4	**9.41 (1.17, 193.30)**	**0.055**
**Perineural invasion**	No	148		
	Yes	9	**6.71 (1.68, 33.12)**	**0.009**
**Invasion beyond subcutaneous tissue**	No	148		
	Yes	9	**4.04 (1.02, 17.09)**	**0.046**
Diameter (cm, log10)		106	2.30 (0.62, 9.39)	0.227
**Thickness >6mm**	No	111		
	Yes	26	**2.27 (0.86, 5.73)**	**0.087**
Ulcer	No	131		
	Yes	26	2.10 (0.84, 5.08)	0.101
**Grading > 1**	No	95		
	Yes	53	**1.97 (0.94, 4.15)**	**0.073**
Margin (cm, log10)		81	1.64 (0.27, 10.39)	0.590
Infundibulocystic features	No	154		
	Yes	3	1.47 (0.07, 15.80)	0.754
Cornu cutaneum	No	154		
	Yes	3	1.47 (0.07, 15.80)	0.754
Keratoakanthoma	No	134		
	Yes	23	0.57 (0.16, 1.65)	0.340

Suppl. Figure 1: Association of clinico-pathological parameters with cSCC progression risk calculated using logistic regression for Cologne patients with available data. Shown are Odds ratios (ORs) with 95% Confidence intervals (CIs) and univariate p-values.

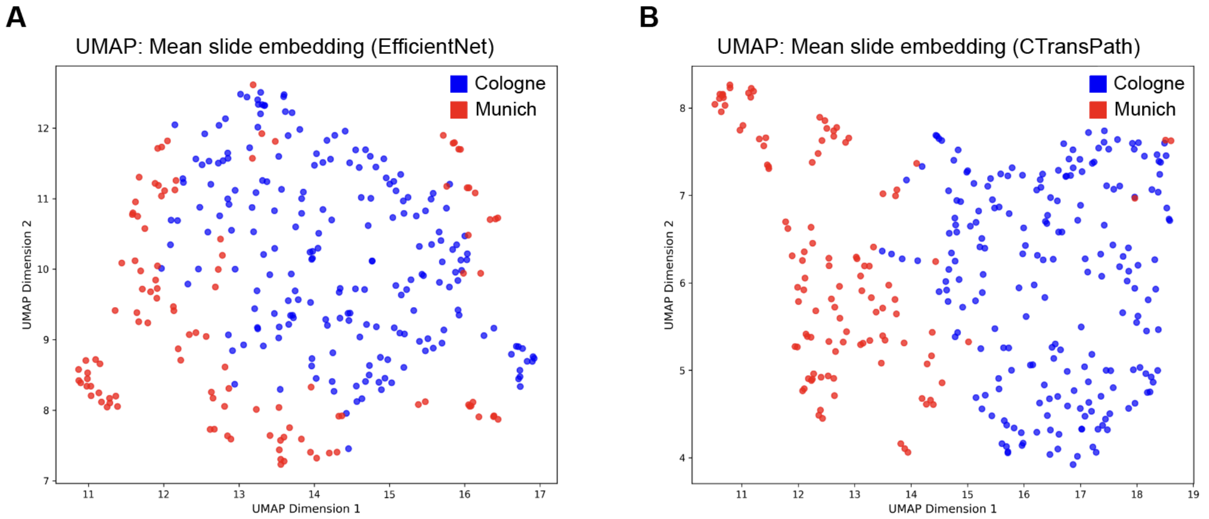

Suppl. Figure 2: UMAP plots of mean slide embeddings of Cologne and Munich. **A**: Computed by the used EfficientNet-v2-L model (trained on the ImageNet dataset of natural images). **B**: Computed by a CTransPath model (trained on a large collection of histopathology slides). Both plots show clear distinction between the two cohorts, indicating systemic differences in image appearance.

A

Variable		N	HR (univ., 95% CI)	p-val (univ.)
**Perineural invasion**	FALSE	148		
	TRUE	9	**3.58 (1.50, 8.56)**	**0.004**
**Vascular invasion**	FALSE	153		
	TRUE	4	**2.71 (0.83, 8.85)**	**0.099**
**Invasion beyond subc. tissue**	FALSE	147		
	TRUE	10	**2.54 (0.99, 6.51)**	**0.052**
Cornu cutaneum	FALSE	154		
	TRUE	3	2.37 (0.32, 17.46)	0.396
**Thickness > 6mm**	FALSE	111		
	TRUE	27	**2.11 (0.97, 4.63)**	**0.061**
Diameter (cm, log10)		104	1.92 (0.61, 5.97)	0.263
Margin (cm, log10)		83	1.88 (0.40, 8.82)	0.425
Grading > 1	FALSE	91		
	TRUE	55	1.68 (0.90, 3.15)	0.104
Ulcer	FALSE	131		
	TRUE	26	1.63 (0.77, 3.44)	0.198
Keratoakanthoma	FALSE	134		
	TRUE	23	0.62 (0.22, 1.74)	0.361

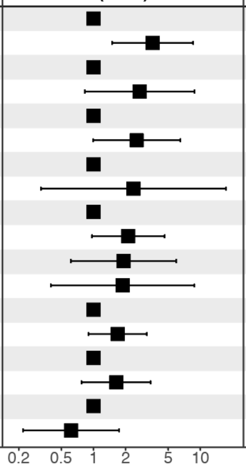

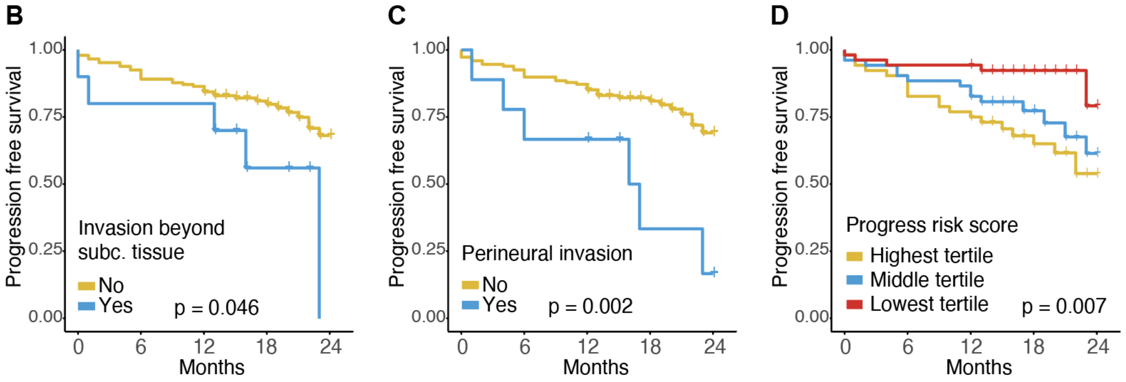

Suppl. Figure 3: A: Univariate association of clinico-pathological parameters derived from medical records & pathology reports with progression-free survival of Cologne cSCC patients. Shown are Hazard ratio (HR) with 95% confidence interval (CI) based on Cox proportional hazard models. N indicates number of patients with available data per category. **B:** Kaplan-Meier curves for Cologne patients with or without invasion beyond subcutaneous tissue. **C:** Kaplan-Meier curves for Cologne patients with or without or perineural invasion. **D:** Progression-free survival of patients grouped into tertiles of the deep learning-based progression risk score. p-values shown in the Kaplan-Meier plots correspond to the log-rank test.

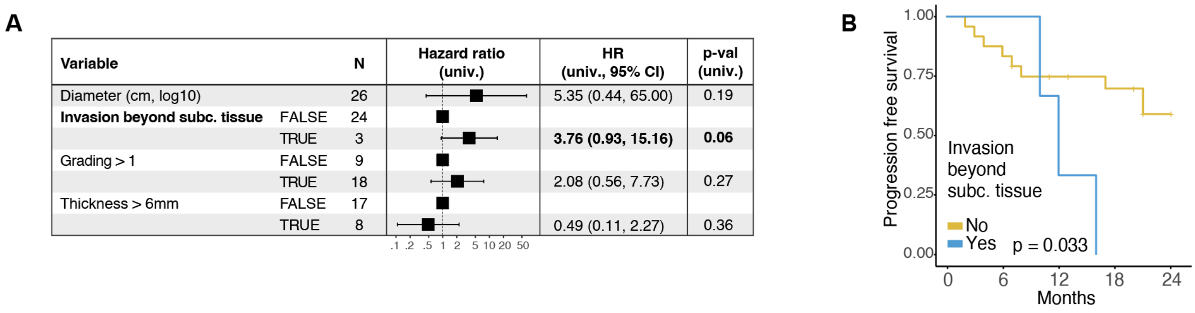

Suppl. Figure 4: A: Univariate association of clinico-pathological parameters derived from medical records & pathology reports with progression-free survival of Bonn cSCC patients. Shown are Hazard ratio (HR) with 95% confidence interval (CI) based on Cox proportional hazard models. N indicates number of patients with available data per category. **B:** Kaplan-Meier curves for Bonn patients with or without invasion beyond subcutaneous tissue.

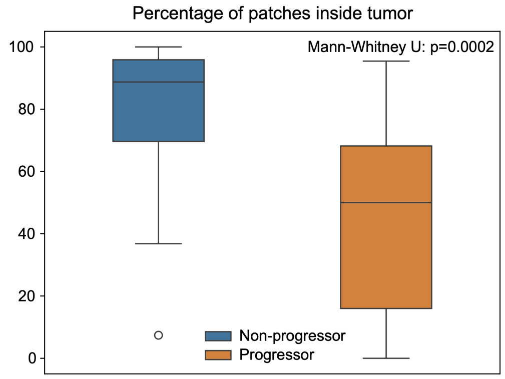

Suppl. Figure 5: Percentage of relevant patches (as detected by IGs) of individual patients inside the tumor regions. On average, non-progressors have more relevant patches inside the tumor compared to progressors.

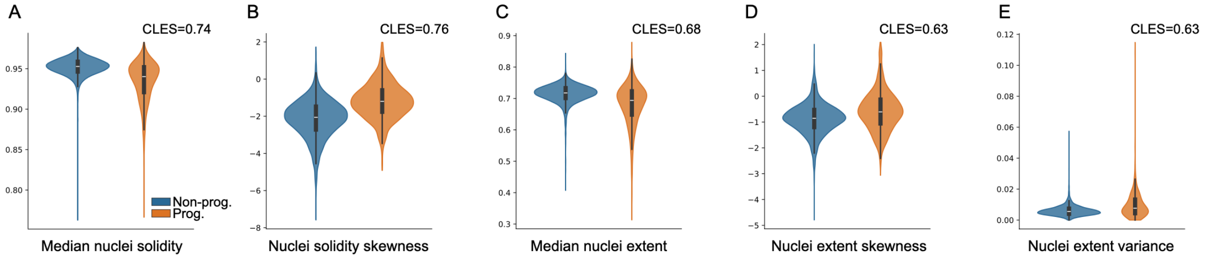

Suppl. Figure 6: Violin plots of 5 tumor cell nuclei morphological features. Non-progressors have larger values of morphological solidity and extent (larger median, negatively-skewed distributions, **A-D**), while morphological extent has a larger variance in tumor cells from progressors **(E)**. All features are significantly different in both groups, with p-values < 0.0001 using Mann-Whitney U test.

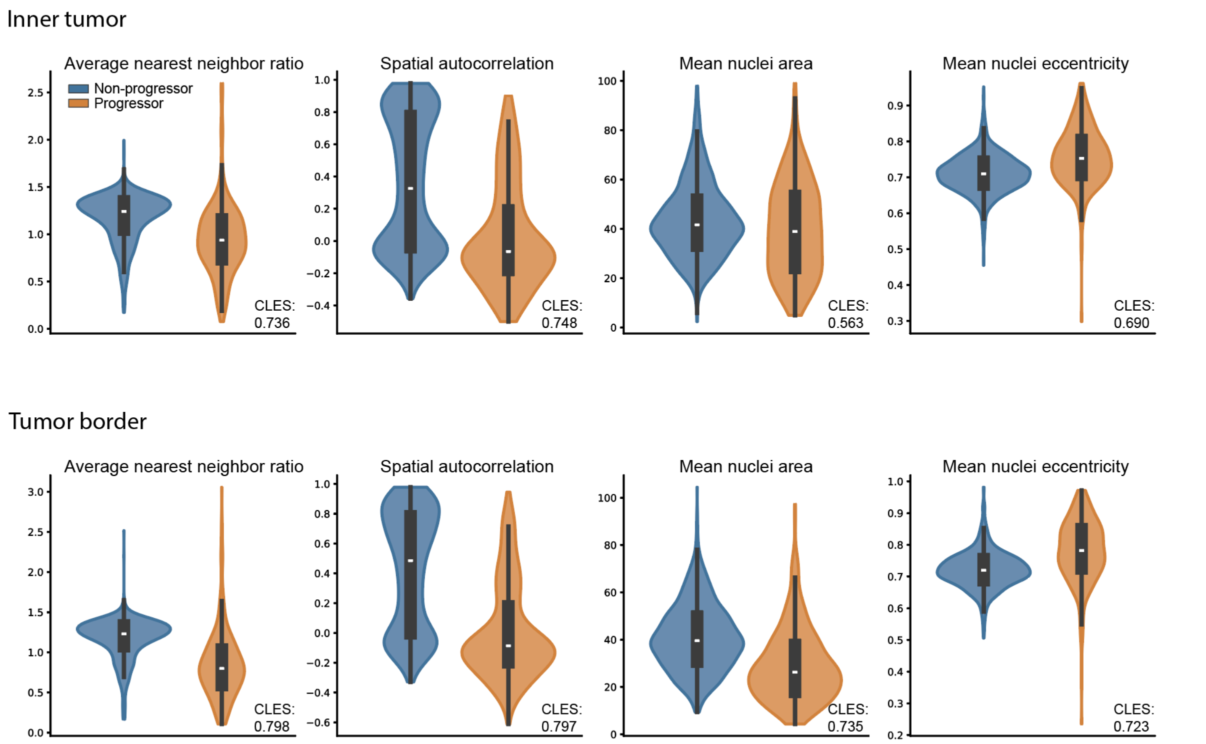

Suppl. Figure 7: Violin plots of the features shown in Fig. 4, computed separately for patches in the tumor border and patches in the inner tumor. We considered the tumor border to be a tissue strip comprising the area closest to the boundary, extending inward approximately 30% of the maximum distance from any tumor pixel to the edge of the tumor, and the inner region to be the remaining central area of the tumor.

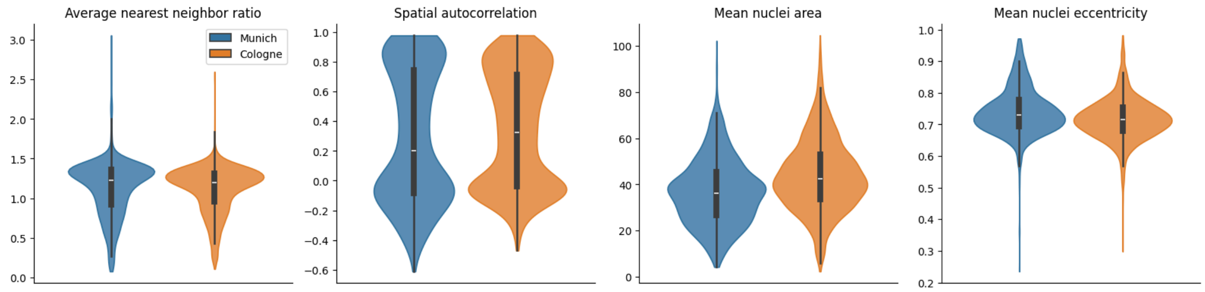

Suppl. Figure 8: Violin plots of the features shown in Fig. 4, grouped by center of origin instead of progression status.

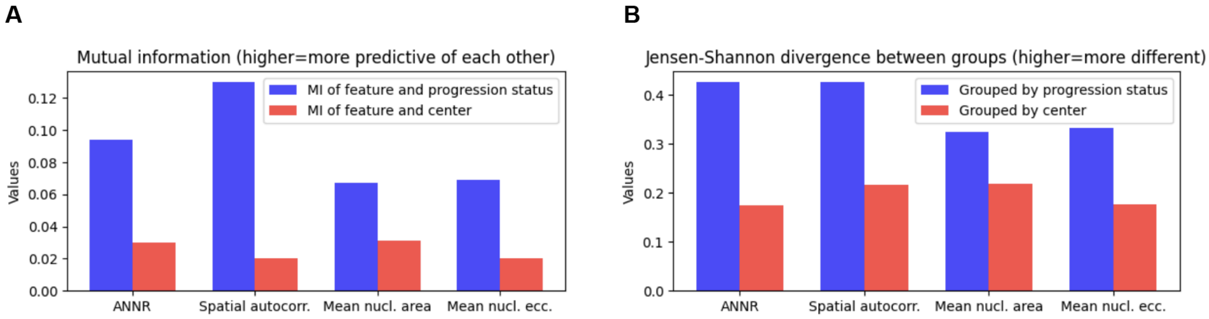

Suppl. Figure 9: Associations between the features shown in the manuscript, with progression status and center of origin, computed for Cologne and Munich. **A**: Mutual information between feature and progression status, and between feature and center. **B**: Jensen-Shannon divergence between groups, when grouped by progression status (progressors vs non-progressors), and when grouped by center (Cologne vs Munich). The features and the progression status are more predictive of each other than the features and the center of origin (**A**). The distributions of progressors and non-progressors are more different than the distributions of Cologne and Munich (**B**).

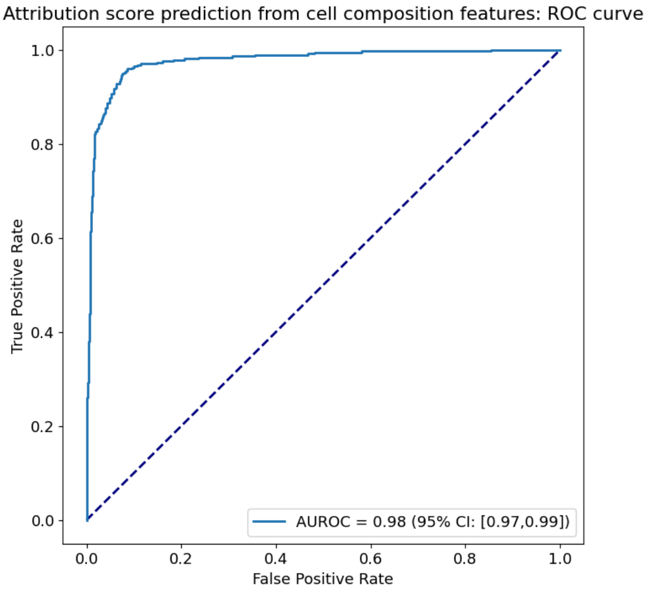

Suppl. Figure 10: ROC curve of the XGBoost patch-level progression status classifier using cell-based features as input.

Suppl. Table 1

Top 100 features with the largest CLES, or probability of superiority, between the groups. To avoid displaying redundant features, pairs of features with a Pearson correlation coefficient bigger than 0.9 are grouped together, and a single feature from the group is shown. The rows are sorted in descending order of CLES for each feature type. The "Higher in" column indicates the group with larger feature values. The fraction of image patches that do not show any value for the features are shown, and features missing in more than 90% of the patches are not displayed. All the features in the table are significantly different in both groups with p<0.0001 using Mann-Whitney U test. Description of nuclei morphology features can be found in the documentation of `skimage.measure` (https://scikit-image.org/docs/stable/api/skimage.measure.html#skimage.measure.regionprops).
